# Advances in BRAF-targeted therapies for non-small cell lung cancer: the promise of encorafenib and binimetinib

**DOI:** 10.1097/JS9.0000000000001051

**Published:** 2024-01-11

**Authors:** Areeba Fareed, Nabiha Amir, Humna Ajaz, Afra Sohail, Rayyan Vaid, Solay Farhat

**Affiliations:** a Department of Medicine, Karachi Medical and Dental College; b Jinnah Sindh Medical University, Karachi, Pakistan; c Faculty of Medical Sciences, Lebanese University, Beirut, Lebanon

Lung cancer (LC) remains to be a paramount global health concern primarily due to its high incidence rates as well as high associated mortality. In fact, it stands as the top cause of cancer-related deaths, making up 18% of all cancer fatalities, and is considered to be the second most frequently diagnosed cancer with a rate of 11.4%^1^. Factors that significantly increase the risk of LC include smoking, secondhand smoke exposure, family history, exposure to carcinogenic chemicals, as well as heavy metals such as radon gas, asbestos, arsenic, chromium, beryllium, and nickel. Additionally, the development of LC can also be influenced by a variety of conditions including pulmonary fibrosis, HIV infection, and alcohol consumption. Notably, men have demonstrated higher rates of incidence and mortality from LC compared to women, which can mainly be attributed to the smoking patterns and baseline exposures^2,3^. Histologically, LC is classically categorized into two main types: small cell lung cancer (SCLC) and non-small cell lung cancer (NSCLC), with NSCLC representing the vast majority of cases, encompassing 85% of all LCs^2^.

In NSCLC patients with advanced disease, up to 69% have mutations in various genes. These include epidermal growth factor receptor (EGFR), anaplastic lymphoma kinase (ALK), c-ros oncogene 1 (ROS1), Kirsten rat sarcoma virus (KRAS), V-raf murine sarcoma oncogene homolog B1 (BRAF), mesenchymal–epithelial transition (MET), human epidermal growth factor receptor (HER2), among others^2^. Often, NSCLC remains undetected until it reaches an advanced stage. Common symptoms include chronic cough, hemoptysis, chest pain, shortness of breath, neck and supraclavicular lymphadenopathy, weight loss, fatigue, fever, and dyspnea^4^. Treatment options of NSCLC include a variety of modalities such as surgery, radiation, chemotherapy, immunotherapy, or molecularly targeted therapy. The patient’s overall health and the disease’s stage ultimately dictate the course and choice of treatment. Patients who are medically stable and have NSCLC in stages I, II, or IIIA (typically when N2 lymph node disease is detected during surgery) need to opt for therapeutic surgical excision. Adjuvant platinum-based chemotherapy is recommended after 5 years for patients in stages II to IIIA, despite serious concerns regarding the high rates of toxicity and recurrence. Additionally, patients with stage III NSCLC often receive combination therapy with both immunotherapy and chemotherapy^5^. In NSCLC, mutations and dysregulation in key components of the MAPK pathway are frequently observed, contributing to tumorigenesis. One of the critical players in this pathway is the BRAF gene, which encodes a protein kinase that is part of the RAF family. BRAF is often mutated in NSCLC, leading to constitutive activation of the MAPK pathway and uncontrolled cell growth. It is estimated that 1–3% of NSCLC cases with adenocarcinoma histology exhibit BRAF mutations, with approximately half having BRAF V600E mutations resulting in a constitutively active BRAF kinase. This mutation leads to continuous signaling through the MAPK pathway, promoting cell proliferation and survival^6^.

Prior efforts to target the MAPK pathway in NSCLC, specifically with BRAF inhibitors alone, encountered limitations, including acquired resistance and inadequate clinical responses. To overcome these challenges, a more comprehensive strategy entails the use of combination therapies, frequently incorporating BRAF inhibitors with MEK inhibitors^7^. Evidence suggests that tumors harboring the BRAF V600E mutation demonstrate aggressive behavior and are associated with a poorer prognosis. Moreover, they often show resistance to platinum-based chemotherapy^8,9^. Thus, the urgency for effective treatment options for these patients is evident. Notably, the Food and Drug Administration (FDA) have previously approved the combination of dabrafenib and trametinib for treating advanced NSCLC in patients with tumors exhibiting the BRAF V600E mutation. This approval was based on the outcomes of two single-arm phase II trials focused on patients with BRAFV600E-mutant NSCLC^10,11^. Common adverse events (AEs) associated with this combination include pyrexia, gastrointestinal symptoms (such as nausea, vomiting, and diarrhea), and constitutional symptoms (like asthenia, decreased appetite, and fatigue). In one of the trials involving previously untreated patients, AEs led to permanent discontinuation in 22% of participants, dose interruption or delay in 75%, and dose reduction in 39% of the patients^10,11^.

On 11 October 2023, the FDA have approved a combination therapy of NSCLC which includes binimetinib (Mektovi, also manufactured by Array BioPharma Inc.) and encorafenib (Braftovi, manufactured by Array BioPharma Inc., a fully owned subsidiary of Pfizer). Adult patients with metastatic NSCLC and BRAF gene mutation were the subject of an open-label, phase II single-arm experiment that served as the basis for this clearance^12^. In this study, to evaluate the efficacy and safety of the combination, 98 patients with BRAFV600E-mutant metastatic NSCLC were enrolled: 59 were treatment-naïve and 39 had been previously treated. The recommended dosages are oral encorafenib 450 mg once daily and binimetinib 45 mg twice daily, taken in 28-day cycles^13^. Results showed that for patients who had not previously received treatment, the disease control rate (DCR) was 64% after 24 weeks, while it was 41% for those who had undergone prior treatment. For those with prior treatment, the median progression-free survival was 9.3 months; however, it was not estimable for treatment-naïve patients. The most common side effects (occurring in ≥25% of patients) included cough, rash, constipation, dyspnea, exhaustion, nausea, diarrhea, musculoskeletal discomfort, vomiting, and stomach pain^13^. Encorafenib has a notably longer dissociation half-life (>30 h) compared to other BRAF inhibitors. This results in prolonged target inhibition and increased potency. In patients treated with dabrafenib plus trametinib, pyrexia was observed in nearly half of the cases. In contrast, pyrexia was less common in patients treated with encorafenib plus binimetinib. Moreover, encorafenib and binimetinib generally have fewer instances of dosage interruption and dose reduction compared to dabrafenib and trametinib. However, it is important to approach these findings with the usual caution associated with cross-trial comparisons^8^.

A thorough examination of the safety and toxicity profiles of dabrafenib/trametinib and encorafenib/binimetinib is crucial for obtaining nuanced insights into their respective clinical advantages. The assessment of adverse effects, long-term consequences, and patient tolerability plays a pivotal role in refining treatment strategies tailored to individual considerations. Furthermore, continuous research is essential to address these limitations and offer a clearer understanding of the safety profiles and comparative efficacy of these regimens. Future investigations should strive to provide more comprehensive data, potentially influencing treatment decisions and optimizing outcomes for patients with NSCLC.

In conclusion, LC remains a major worldwide health concern, with its high mortality rates being linked to a variety of risk factors. NSCLC dominates LC diagnoses, and the emergence of targeted therapies, particularly for specific genetic alterations like BRAF V600E, holds promise for the future. The recent FDA approval of encorafenib and binimetinib for BRAF-altered NSCLC signifies a positive trajectory, demonstrating improved patient outcomes and a favorable safety profile compared to existing treatments. The examination of resistance mechanisms to BRAF/MEK inhibitors is crucial, uncovering common obstacles such as secondary mutations and alternative pathway activation. These challenges underscore the necessity for refined therapeutic strategies, guiding future research for more effective interventions in the dynamic landscape of NSCLC treatment. Given the growing significance of immune checkpoint inhibitors (ICIs) in cancer treatment, the exploration of potential combinations or sequencing of encorafenib/binimetinib with ICIs becomes paramount. Synergies between targeted therapies and immunotherapy may enhance treatment efficacy, offering a promising approach to overcome resistance and improve outcomes. Investigating these combined approaches has the potential to revolutionize treatment paradigms for BRAF-mutant NSCLC, addressing limitations and maximizing therapeutic benefits^14^. This progress underscores the increasing focus on tailored treatments in the fight against LC, emphasizing the continuous effort to enhance treatment approaches and boost both the life expectancy and life quality of those impacted.

## Ethical approval

Not applicable.

## Consent

Not applicable.

## Sources of funding

The authors did not receive any financial support for this work. No funding has been received to conduct this study.

## Author contribution

All authors have contributed equally to this work.

## Conflicts of interest disclosure

There are no conflicts of interest.

## Research registration unique identifying number (UIN)

Not applicable.

## Guarantor

Solay Farhat, MD, Faculty of Medical Sciences, Lebanese University, Beirut, Lebanon; e-mail: solayfarhat@gmail.com; ORCID: 0000-0002-5108-7658.

## Data availability statement

Not applicable.

## Provenance and peer review

Not applicable.

## References

[1] Neumann JM Freitag H Hartmann JS. Subtyping non-small cell lung cancer by histology-guided spatial metabolomics. J Cancer Res Clin Oncol 2022;148:351–360. 34839410 10.1007/s00432-021-03834-w PMC8800912

[2] Fois SS Paliogiannis P Zinellu A. Molecular epidemiology of the main druggable genetic alterations in non-small cell lung cancer. Int J Mol Sci 2021;22:612. 33435440 10.3390/ijms22020612 PMC7827915

[3] Stabellini N Bruno DS Dmukauskas M. Sex differences in lung cancer treatment and outcomes at a large hybrid academic-community practice. JTO Clin Res Rep 2022;3:100307. 35400080 10.1016/j.jtocrr.2022.100307 PMC8983352

[4] Xing PY Zhu YX Wang L. What are the clinical symptoms and physical signs for non-small cell lung cancer before diagnosis is made? A nation-wide multicenter 10-year retrospective study in China. Cancer Med 2019;8:4055–4069. 31150167 10.1002/cam4.2256 PMC6639195

[5] Alduais Y Zhang H Fan F. Non-small cell lung cancer (NSCLC): a review of risk factors, diagnosis, and treatment. Medicine (Baltimore) 2023;102:e32899. 36827002 10.1097/MD.0000000000032899 PMC11309591

[6] Tsamis I Gomatou G Chachali SP. BRAF/MEK inhibition in NSCLC: mechanisms of resistance and how to overcome it. Clin Transl Oncol 2023;25:10–20. 35729451 10.1007/s12094-022-02849-0

[7] Rivera-Concepcion J Uprety D Adjei AA. Challenges in the use of targeted therapies in non-small cell lung cancer. Cancer Res Treat 2022;54:315–329. 35209703 10.4143/crt.2022.078 PMC9016301

[8] Stinchcombe TE. Encorafenib and binimetinib: a new treatment option for BRAFV600E-mutant non–small-cell lung cancer. J Clin Oncol 2023;41:3679–3681. 37270690 10.1200/JCO.23.00983

[9] Riely GJ Ahn MJ Felip E. Encorafenib plus binimetinib in patients with BRAFV600-mutant non-small cell lung cancer: phase II PHAROS study design. Future Oncol Lond Engl 2022;18:781–791. 10.2217/fon-2021-1250 34918546

[10] Planchard D Smit EF Groen HJM. Dabrafenib plus trametinib in patients with previously untreated BRAFV600E-mutant metastatic non-small-cell lung cancer: an open-label, phase 2 trial. Lancet Oncol 2017;18:1307–1316. 28919011 10.1016/S1470-2045(17)30679-4

[11] Planchard D Kim TM Mazieres J. Dabrafenib in patients with BRAF(V600E)-positive advanced non-small-cell lung cancer: a single-arm, multicentre, open-label, phase 2 trial. Lancet Oncol 2016;17:642–650. 27080216 10.1016/S1470-2045(16)00077-2 PMC5006181

[12] U.S. Food & Drug Administration. FDA approves encorafenib with binimetinib for metastatic non-small cell lung cancer with a BRAF V600E mutation, 10 November 2023. Accessed 23 October 2023. https://www.fda.gov/drugs/resources-information-approved-drugs/fda-approves-encorafenib-binimetinib-metastatic-non-small-cell-lung-cancer-braf-v600e-mutation

[13] Riely GJ Smit EF Ahn MJ. Phase II, Open-label study of encorafenib plus binimetinib in patients with BRAFV600-mutant metastatic non-small-cell lung cancer. J Clin Oncol 2023;41:3700–3711. 37270692 10.1200/JCO.23.00774

[14] Mountzios G Remon J Hendriks LEL. Immune-checkpoint inhibition for resectable non-small-cell lung cancer – opportunities and challenges. Nat Rev Clin Oncol 2023;20:664–677. 37488229 10.1038/s41571-023-00794-7

